# A multidimensional comparison of ChatGPT, Google Translate, and DeepL in Chinese tourism texts translation: fidelity, fluency, cultural sensitivity, and persuasiveness

**DOI:** 10.3389/frai.2025.1619489

**Published:** 2025-07-24

**Authors:** Shiyue Chen, Yan Lin

**Affiliations:** ^1^School of Humanities and Foreign Languages, Zhejiang Shuren University, Hangzhou, China; ^2^School of Foreign Languages, Ningxia Normal University, Guyuan, China

**Keywords:** tourism, tourism translation, machine translation, ChatGPT, prompt design assessing

## Abstract

This study systematically compares the translation performance of ChatGPT, Google Translate, and DeepL on Chinese tourism texts, focusing on two prompt-engineering strategies. Using a mixed-methods approach that combines quantitative expert assessments with qualitative analysis, the evaluation centers on fidelity, fluency, cultural sensitivity, and persuasiveness. ChatGPT outperformed its counterparts across all metrics, especially when culturally tailored prompts were used. However, it occasionally introduced semantic shifts, highlighting a trade-off between accuracy and rhetorical adaptation. Despite its strong performance, human post-editing remains necessary to ensure semantic precision and professional standards. The study demonstrates ChatGPT’s potential in domain-specific translation tasks while calling for continued oversight in culturally nuanced content.

## 1 Introduction

Machine translation has become an essential resource in an increasingly globalized world. The heightened demand for cross-linguistic communication among individuals, businesses, organizations, and governmental bodies underscores its strategic importance (Brynjolfsson et al., 2019; Liu et al., 2022). Across various sectors—including international trade, diplomacy, education, academic research, and tourism—machine translation facilitates the seamless exchange of information and ideas, connecting people from diverse linguistic and cultural backgrounds (Rivera-Trigueros, 2022).

The development of machine translation systems has long been a central focus of both linguistic and computational research. Recent breakthroughs in artificial intelligence (AI) and machine learning (ML), particularly the introduction of transformer architectures (Vaswani et al., 2017), alongside advances in computational power, have led to the emergence of large language models (LLMs). These are powerful tools for processing human language with markedly improved accuracy and context awareness (Freitag et al., 2021). Nonetheless, challenges persist in specialized domains such as tourism, where texts often demand precise translations that reflect cultural nuances, idiomatic expressions, and regional language variations. Brochures, travel guides, promotional materials, and websites not only convey factual information but also employ persuasive language and culturally appropriate expressions (Turra, 2020). This dual role underscores the importance of capturing both the literal meaning and the underlying cultural nuances, emotional appeal, and marketing objectives embedded in the source text. Consequently, tourism translation necessitates a careful balance between accuracy and cultural adaptation to ensure the translated content resonates effectively with target audiences (Metwally and Asiri, 2023).

China presents a compelling case for studying tourism-related machine translation due to its rich cultural heritage, linguistic complexity, and rapidly growing global tourism sector (Li et al., 2021). As one of the world’s most visited countries, China consistently attracts a significant number of international visitors each year (Gao et al., 2023). Chinese tourism materials frequently encompass deeply embedded cultural references, idiomatic expressions, historical narratives, and promotional rhetoric designed to appeal to a global audience unfamiliar with Chinese traditions and contexts. Therefore, accurately translating these materials demands sophisticated linguistic and cultural adaptations that conventional machine translation systems may struggle to achieve.

This study addresses these complexities by evaluating the performance of ChatGPT—a leading large language model—in comparison to two widely utilized conventional machine translation tools, Google Translate and DeepL Translator. While previous research has examined machine translation’s (MT) efficacy across various domains, limited studies have explored how LLM-driven translation performs specifically in tourism contexts, where cultural adaptation and persuasive language are crucial. Specifically, the research examines translations of Chinese tourism materials into English, focusing on four key dimensions: fidelity, fluency, cultural adaptation, and persuasiveness. By systematically analyzing and comparing outputs across these dimensions, the study aims to identify the respective strengths and limitations of these systems when confronted with the linguistic and cultural intricacies inherent in tourism translations. The findings will contribute to a deeper understanding of the efficacy of modern LLMs relative to traditional machine translation systems in this specialized domain.

## 2 Literature review

Machine translation (MT) has become indispensable for overcoming linguistic barriers across a variety of domains (Chatzikoumi, 2020). Recent advancements in artificial intelligence (AI)—particularly deep learning and neural network approaches—have significantly enhanced MT systems, culminating in the emergence of neural machine translation (NMT). By harnessing large parallel corpora, NMT models learn semantic and syntactic features of languages, allowing them to produce translations that are notably more fluent, accurate, and natural than earlier rule-based or statistical methods. Studies by Naveen and Trojovský (2024) and Dwivedi et al. (2025) further highlight the transformative impact of NMT on translation quality, emphasizing its capacity to handle complex linguistic structures and generate outputs that approach human-like fluency. A pivotal development in this field is the introduction of LLMs, exemplified by ChatGPT, which was developed by OpenAI. Built on the Generative Pre-trained Transformer (GPT) architecture, ChatGPT draws upon extensive textual datasets to deliver coherent, contextually appropriate, and highly fluent outputs (Bansal et al., 2024). Unlike traditional MT systems that focus primarily on language conversion, ChatGPT’s broader language understanding enables it to adapt to specialized tasks.

Tourism texts—encompassing brochures, travel guides, promotional materials, and websites—present unique challenges for machine translation, primarily due to their reliance on persuasive language, idiomatic expressions, and culturally embedded references. Translating these materials effectively requires a delicate balance among four core dimensions. Fidelity ensures the accurate conveyance of the source text’s meaning and intent, thus reinforcing the reliability and trustworthiness of promotional content (Sulaiman, 2016; Qassem and Sahari, 2024). Nonetheless, achieving fidelity in tourism texts can be particularly demanding, given their frequent use of idiomatic expressions, metaphorical language, and culturally specific terms that often lack direct equivalents in other languages. Fluency likewise proves critical, as producing natural, grammatically sound translations is vital for maintaining reader engagement and credibility (Carvalho et al., 2023). Additionally, cultural sensitivity promotes alignment with the values, norms, and expectations of the target audience, thereby bridging cultural divides and preventing misunderstandings or alienation (Sumberg, 2004; Viken et al., 2021). Finally, persuasiveness stands as a defining attribute of tourism materials, reflecting their inherently promotional aims. This dimension underscores the capacity of a translation to inspire and motivate readers, retaining the source text’s emotional and rhetorical appeal to attract international travelers. These four dimensions—fidelity, fluency, cultural sensitivity, and persuasiveness—constitute the evaluative framework of the present study and have been selected based on their prominence in prior literature as critical success factors in tourism translation. Crucially, these dimensions are central to post-editing (PE) research in promotional genres, where the goal is not merely to produce an adequate translation but a publishable one that meets specific marketing objectives. As studies on the translation of promotional content show, achieving this level of quality almost invariably requires human intervention, typically in the form of full post-editing, to address the very nuances that automated systems struggle with (Jakobsen, 2018; Mah, 2020).

Previous studies have largely concentrated on general translation tasks or broad linguistic evaluations (Moreno-Romero and Hermán-Carvajal, 2024; Rosa-Sorlozano and Candel-Mora, 2025), neglecting specialized domains that demand nuanced cultural and rhetorical adaptation, especially within the context of Chinese-English translation. Furthermore, while some research has examined the strengths of NMT systems in producing grammatically accurate and fluent translations (Rosa-Sorlozano and Candel-Mora, 2025), insufficient attention has been paid to their limitations in capturing idiomatic expressions, persuasive elements, and culturally specific references that are pivotal in tourism communication (Li et al., 2022; Chen and Zhou, 2024). Additionally, earlier evaluations predominantly focused on European language pairs, with limited empirical inquiry into Chinese-English translation contexts. This gap is particularly salient from a post-editing perspective, as the cognitive effort and types of edits required can vary significantly depending on the genre and language pair. Understanding the specific weaknesses of MT for Chinese tourism texts is therefore a prerequisite for developing effective human-in-the-loop workflows. This study addresses this gap by offering a domain-specific and language-pair-specific evaluation.

MT systems have struggled to address the distinctive demands of tourism texts. Early approaches, such as rule-based and statistical MT models, relied heavily on literal translations, often failing to capture the idiomatic expressions and cultural nuances integral to tourism content (Macketanz et al., 2017; Wang, 2022). Consequently, these systems frequently produced outputs that appeared awkward, mechanical, and ultimately unconvincing for target audiences. Although the advent of NMT systems has markedly improved fluency and grammatical accuracy, such advancements remain insufficient for the complex requirements of tourism texts. Empirical studies indicate that traditional NMT systems tend to prioritize formal accuracy and syntactic correctness at the expense of creative and rhetorical adaptation, thereby diminishing the persuasive and culturally rich elements of tourism materials (Barreiro et al., 2014; Jibreel, 2023). As a result, these systems often generate translations that, while technically accurate, lack the emotional resonance and cultural appropriateness necessary to engage readers effectively. This shifts the nature of the post-editing task: instead of primarily correcting grammatical errors, the human translator must engage in substantial creative rewriting and cultural adaptation to restore the text’s persuasive function.

Unlike traditional NMT systems, ChatGPT adopts a decoder-only architecture that synthesizes contextual and linguistic cues, enabling the generation of nuanced, contextually relevant, and stylistically refined outputs. The model’s capacity to deliver fluent, coherent, and natural-sounding translations has been widely acknowledged (Khan et al., 2023; Shamim and Mallik, 2024). A salient advantage of ChatGPT is its ability to perform in-context learning, wherein examples embedded within prompts guide the model to produce translations tailored to specific requirements (Gao Y. et al., 2024). From a post-editing standpoint, this functionality can be conceptualized as a form of pre-editing, where carefully crafted prompts are used to generate a higher-quality initial draft, thereby aiming to reduce subsequent PE effort. This functionality is especially critical in the tourism domain, where well-crafted prompts can highlight cultural sensitivity, rhetorical effectiveness, or stylistic nuances unique to tourism discourse (Remountakis et al., 2023). This connection provides the theoretical basis for the current research, which investigates whether tailored prompts can enhance ChatGPT’s translation performance in tourism contexts.

Furthermore, the extensive training of ChatGPT on large and heterogeneous datasets strengthens its ability to handle culturally specific elements, such as idiomatic expressions (Zhong et al., 2024; AlAfnan, 2025) and references to local customs or cuisines (Pawar et al., 2024), which often form the core of tourism texts. By leveraging its broad cultural knowledge, ChatGPT can reinterpret culturally embedded phrases and adapt them into contextually resonant equivalents for target audiences. Rather than defaulting to overly literal translations that risk alienating readers or misrepresenting the essence of the source text, ChatGPT can produce culturally sensitive and emotionally engaging outputs. Its capacity to render idiomatic expressions in a manner that preserves their emotional and cultural significance underscores its potential to bridge linguistic and cultural gaps—an observation aligned with Bhattacharya et al. (2024), who highlight ChatGPT’s efficacy in creative and persuasive tasks.

By contrast, conventional NMT systems such as Google Translate and DeepL Translator, while effective for many general translation tasks, often prove less adept at addressing the rhetorical and cultural demands inherent in tourism texts. Their reliance on encoder-decoder architectures frequently prioritizes grammatical accuracy and fluency (Barreiro et al., 2014; Jibreel, 2023). Although this focus may adequately facilitate direct linguistic conversions, it often inadequately adapts culturally nuanced content or persuasive language—both of which are crucial for successfully engaging international tourism audiences. These comparative limitations set the stage for the current research, which explores the relative advantages and constraints of ChatGPT vis-à-vis Google Translate and DeepL in tourism translation. In light of these challenges and opportunities, the present study seeks to address the following research questions:

1. How do Google Translate, DeepL Translator, and ChatGPT perform in terms of fidelity, fluency, cultural sensitivity, and persuasiveness when translating Chinese tourism materials into English?
2. To what extent can tailored prompts enhance ChatGPT’s performance in tourism translation?
3. What are the advantages and limitations of ChatGPT compared to Google Translate and DeepL Translator in tourism translation?

## 3 Methodology

A mixed-methods research design was adopted, integrating expert-led quantitative evaluations with qualitative analyses of the translated excerpts.

### 3.1 Materials

This study selected 20 Chinese tourism texts—averaging 150–200 words each—from official promotional materials, travel guides, tourism websites, and destination brochures to minimize potential overlap with ChatGPT’s training data and enhance assessment validity. These texts can be found in the Supplementary material. The 20 texts, characterized by a blend of informational content and culturally persuasive language, provide an ideal basis for evaluating translation quality in terms of fidelity, fluency, cultural sensitivity, and persuasiveness. While a larger sample size may be desirable, 20 texts were chosen to ensure a focused, manageable dataset for in-depth analysis, while still capturing a diverse range of content types. The selected texts, which represent a variety of promotional and informative materials within the tourism domain, reflect key features—such as cultural nuance, persuasive tone, and informational density—that are relevant for assessing translation quality. Given that the goal is to evaluate the translation’s capacity to handle both linguistic and cultural nuances in a typical tourism context, this sample is sufficient to draw meaningful conclusions about the core dimensions of fidelity, fluency, and cultural sensitivity. Future work can expand the dataset to include a broader range of genres, as suggested, to further validate and extend the findings. The translation performance of three systems was compared: Google Translate, DeepL Translator, and ChatGPT (GPT-3.5). Google Translate (accessed via its public web interface) supports over 130 languages, while DeepL (accessed via its public web interface, the free version), introduced in 2017 and noted for its high-quality outputs, currently covers 31 languages. ChatGPT, released by OpenAI in 2022, was evaluated using two distinct prompts applied to the GPT-3.5 model accessed via the OpenAI API. To examine ChatGPT’s performance in translating Chinese tourism texts, two distinct prompts were employed:

*Prompt 1*: “Please provide the English translation for the following text.”

*Prompt 2*: “The following text is a Chinese tourism description. First, carefully interpret the meaning, and then translate it into fluent, culturally sensitive, and persuasive English suitable for international readers.”

Prompt 1 was deliberately kept straightforward, offering minimal contextual or stylistic guidance. This design followed earlier methodologies, such as those described by Gao Y. et al. (2024), where a basic prompt is used to assess ChatGPT’s general translation capabilities. In contrast, Prompt 2 was developed in line with principles of task-specific and domain-specific prompting (Kepel and Valogianni, 2024). By providing explicit instructions pertinent to the tourism domain, Prompt 2 aimed to guide ChatGPT’s focus on interpretation, fluency, cultural adaptation, and persuasiveness, all of which are critical for effective tourism translations. In developing these prompts, preliminary pilot testing was conducted to determine the most effective phrasing. Various prompt formulations were trialed, including those emphasizing “no omissions” or providing brief in-context examples, to gauge their impact on translation quality. After careful evaluation, it was determined that Prompt 1’s simplicity and general approach allowed for an unbiased assessment of ChatGPT’s translation capabilities, while Prompt 2’s specific instructions better suited the task of evaluating culturally sensitive and persuasive translations. Moreover, Prompt 2 incorporated concepts of in-context learning and chain-of-thought reasoning (Yu et al., 2023; Cheng et al., 2024), encouraging ChatGPT to reflect on the source content’s meaning prior to producing the final translation. While these two prompts were found to be effective based on initial testing, future studies could explore further variations, such as incorporating “few-shot” examples, to refine and optimize prompt performance.

### 3.2 Procedures

In this study, seven native Chinese speakers—each holding a PhD in English Studies and possessing at least 5 years of relevant professional experience—were recruited as expert evaluators to assess the translation quality. The recruitment period for this study commenced on December 13, 2024, and concluded on December 27, 2024. While the expertise of the evaluators in both Chinese and English ensures a deep understanding of fidelity to the original Chinese texts, it is important to acknowledge a potential bias due to their native Chinese background. Specifically, Chinese evaluators may have different intuitions about “fluency” and “cultural appropriateness” in the translations compared to native English speakers, especially when assessing cultural sensitivity. Since cultural markers can vary between Chinese and Western perspectives, there is a possibility that the evaluators might prioritize elements that are more familiar or relevant to a Chinese audience. This bias may influence how the translations are rated, particularly about how “culturally sensitive” they appear to the intended target audience. While incorporating a small cohort of native English evaluators could indeed help to capture the perspectives of non-Chinese readers, the decision was made to focus solely on Chinese evaluators for this study, as the primary objective was to assess translations from the viewpoint of native Chinese speakers engaging with the content in their cultural context. Future research may consider integrating native English evaluators to further explore how Western readers perceive fluency and persuasiveness in the translations. The evaluators applied five predefined criteria—fidelity, fluency, cultural sensitivity, persuasiveness, and overall performance—using a 5-point Likert scale (1 = Very Low, 5 = Excellent). As shown in Table 1, each criterion was explicitly defined to ensure consistency in application and clarity in interpretation. These dimensions were selected based on their alignment with established translation quality assessment frameworks, and their particular relevance to the tourism domain. Fidelity and fluency are core components in virtually all translation evaluation protocols (Rennert, 2010; Han et al., 2021), reflecting the semantic accuracy and naturalness of the target text. Cultural sensitivity and persuasiveness, while less commonly included in general-purpose evaluation rubrics, are critical in tourism translation, where conveying local color, engaging the audience, and maintaining promotional intent are essential communicative goals (Sulaiman, 2016; Viken et al., 2021; Alhaj, 2024). Thus, the chosen dimensions reflect both theoretical foundations and domain-specific requirements. The overall performance rating was synthesized from the evaluators’ judgments across the four primary dimensions (fidelity, fluency, cultural sensitivity, and persuasiveness).

**Table 1:** Rating scales.

Fidelity
The extent to which the translation accurately preserves the meaning, intent, and details of the source text, avoiding omissions or misrepresentations.
1. Very low: captures <20% of the original meaning and content, with significant distortions.
2. Low: retains only a limited portion of the original meaning and frequently misrepresents details.
3. Moderate: conveys the general meaning but includes some inaccuracies or omissions.
4. Good: accurately reflects the meaning and intent of the original text, with only minor omissions or errors.
5. Excellent: faithfully preserves the meaning and content of the source text in its entirety, with no distortions or omissions.
Fluency
The readability, grammatical accuracy, and natural flow of the translated text in the target language.
1. Very low: highly disjointed, with numerous grammatical errors and awkward phrasing.
2. Low: contains several grammatical inconsistencies and unnatural sentence structures, reducing clarity.
3. Moderate: generally coherent but with occasional grammatical mistakes or awkward wording.
4. Good: smooth and coherent, with few grammatical errors and a mostly natural flow.
5. Excellent: reads fluently and naturally, with no grammatical errors or awkward expressions.
Cultural sensitivity
The degree to which the translation adapts cultural references, idioms, and stylistic elements to align with the norms and expectations of the target audience.
1. Very low: fails to adapt any cultural elements, resulting in a translation that feels out of context or inappropriate.
2. Low: adapts only minimal cultural references, with most remaining untranslated or awkwardly presented.
3. Moderate: adapts some cultural elements but misses key references or nuances important to the audience.
4. Good: effectively adapts most cultural references and stylistic elements, making the text relatable to the target audience.
5. Excellent: skillfully incorporates cultural nuances and idiomatic expressions, ensuring the translation resonates fully with the target audience.
Persuasiveness
The effectiveness of the translation in retaining the original text’s ability to appeal to and influence the target audience.
1. Very low: completely lacks the original text’s emotional appeal or persuasive intent.
2. Low: retains only a minimal degree of the original promotional tone or emotional impact.
3. Moderate: maintains some persuasive elements but lacks consistency in tone or emotional engagement.
4. Good: retains most of the original text’s persuasive tone and emotional appeal, engaging the reader effectively.
5. Excellent: fully preserves the persuasive and emotive qualities of the source text, making the translation compelling and impactful.
Overall performance
A holistic assessment that considers fidelity, fluency, cultural sensitivity, and persuasiveness.
1. Very low: translation quality is poor across all criteria, failing to meet basic standards.
2. Low: the translation meets minimal standards but is significantly flawed in multiple areas.
3. Moderate: adequate performance across most criteria but with room for noticeable improvement.
4. Good: high-quality translation that effectively balances fidelity, fluency, cultural sensitivity, and persuasiveness, with only minor issues.
5. Excellent: exceptional translation that excels in all criteria, providing a seamless and professional output.

To address the potential subjectivity in the rating of fidelity, especially in cases where translations involve embellishments or persuasive adaptations, raters were provided with concrete examples of both acceptable and unacceptable “interpretive liberties.” These examples clarified the boundary between minor, acceptable adaptations—such as rephrasing for fluency or adding context for clarity—and more substantial distortions that could alter the original meaning or intent. However, in the absence of clear-cut guidelines for all possible scenarios, there may still have been some disagreement among raters on where to draw the line between acceptable adaptation and misrepresentation. To mitigate this, any discrepancies in ratings were discussed to ensure consensus on how to categorize specific translations. Despite these measures, it is acknowledged that fidelity ratings may still encompass some degree of inter-rater disagreement, particularly when the line between acceptable adaptation and distortion is not immediately clear. Future studies could aim to further refine the guidelines for fidelity assessment and explore how training raters on these nuances might improve consistency in such evaluations.

To facilitate the evaluation, the 20 Chinese tourism texts and their 80 English translations—produced by Google Translate, DeepL Translator, and ChatGPT (via Prompts 1 and 2)—were compiled into an Excel spreadsheet. Each of the seven evaluators received a personalized version of the file, in which both the source texts and the translations were presented in a unique randomized order to minimize order effects and reduce potential bias. Translations for each source text were grouped together but anonymized, so that the evaluators remained unaware of which system had generated each version, thereby ensuring a blind review. Evaluators were instructed to assess each translation across five predefined dimensions—fidelity, fluency, cultural sensitivity, persuasiveness, and overall performance—based on clearly defined criteria (see Table 1). All evaluators rated the complete set of 80 translations, and entered their scores into their respective Excel files, which were then returned to the authors. The collected data were subsequently aggregated and analyzed to generate the final evaluation results. This structured procedure ensured a systematic, unbiased, and replicable evaluation process.

### 3.3 Informed consent statement

All participants provided written informed consent before their participation. The consent form included comprehensive information about the study’s objectives, procedures, potential benefits, and the voluntary nature of participation. It also highlighted participants’ right to withdraw from the study at any time without facing any consequences, the measures taken to ensure confidentiality and anonymity, and a summary of the anticipated outcomes of the study. The research was carried out in accordance with the Helsinki Declaration guidelines and was approved by the Ethics Committee of the School of Humanities and Foreign Languages at Zhejiang Shuren University.

## 4 Findings

The data for this study were initially provided in Comma-Separated Values (CSV) format and were subsequently loaded into a MySQL relational database (version 8.4 LTS) for management. A computational pipeline was developed in Python (version 3.9) to perform all data extraction, processing, and analysis tasks. The core software environment for this pipeline consisted of the NumPy (v2.0.2), Pandas (v2.2.3), and Seaborn (v0.13.2) packages, ensuring that the translation evaluation was both robust and fully replicable. Five linear mixed-effects regression models—corresponding to fidelity, fluency, cultural sensitivity, persuasiveness, and overall performance—were constructed using the *statsmodels* package. The translation system was treated as a fixed effect, with two ChatGPT (CP) prompt conditions (CP1 and CP2), DeepL Translator (DL), and Google Translate (GT) as levels. Raters and source texts were included as random effects to account for individual and contextual variability. This mixed-effects modeling approach effectively captured the crossed structure of the data—multiple raters assessing multiple texts—thereby providing a more accurate estimation of each system’s translation quality (see Tables 2–7). In addition to determining the statistical significance of our findings via *p*-values, we assess their practical significance by calculating effect sizes. This dual approach allows for a more nuanced interpretation of the results, distinguishing between differences that are merely statistical artifacts and those that represent meaningful gaps in performance. For this purpose, we employ Cohen’s d, a standardized measure of the mean difference between two groups. The magnitude of Cohen’s d is critically dependent on the pooled standard deviation of the compared groups ( $d=(M_{1}-M_{2})/SD_{\text{pooled}}\;$ ). This implies that even a small mean difference on our 5-point evaluation scale can yield a large effect size if the variance in the data is low. Such a low-variance scenario is common in evaluations with clear criteria and high rater agreement, or when assessing systems that exhibit highly consistent behavior. Consequently, as we present our results, findings of a large effect size—even those derived from a small raw score difference—should be understood to reflect a stable, reliable, and practically important performance gap between the evaluated systems.

**Table 2:** Mean and standard deviation of scores.

MT system	Fidelity	Fluency	Cultural sensitivity	Persuasiveness	Overall performance
CP1	4.04 (0.19)	3.79 (0.20)	3.77 (0.18)	4.04 (0.26)	3.87 (0.15)
CP2	3.69 (0.22)	4.23 (0.29)	4.09 (0.20)	4.39 (0.20)	4.07 (0.32)
DL	2.34 (0.26)	2.29 (0.23)	2.07 (0.31)	2.16 (0.28)	2.20 (0.18)
GT	1.93 (0.21)	2.13 (0.32)	1.84 (0.17)	1.89 (0.16)	1.90 (0.29)

**Table 3:** Fidelity parameter.

Fidelity	Mean score	*β*	SE	*t*	df	*p*
CP1 vs. DL	4.04 vs. 2.34	0.6856	0.117	5.843	5	0.002
CP1 vs. GT	4.04 vs. 1.93	0.8955	0.057	15.663	5	<0.001
DL vs. GT	2.34 vs. 1.93	1.1500	0.198	5.813	5	0.002
CP2 vs. DL	3.69 vs. 2.34	0.8054	0.128	6.293	5	0.001
CP2 vs. GT	3.69 vs. 1.93	0.9955	0.154	6.475	5	0.001
CP1 vs. CP2	4.04 vs. 3.69	0.8156	0.120	6.771	5	<0.001

**Table 4:** Fluency parameter.

Fluency	Mean score	*β*	SE	*t*	df	*p*
CP1 vs. DL	3.79 vs. 2.29	0.7558	0.177	4.260	5	0.008
CP1 vs. GT	3.79 vs. 2.13	0.5895	0.060	9.756	5	<0.001
DL vs. GT	2.29 vs. 2.13	0.6031	0.166	3.628	5	0.015
CP2 vs. DL	4.23 vs. 2.29	1.0388	0.310	3.356	5	0.020
CP2 vs. GT	4.23 vs. 2.13	0.7957	0.173	4.611	5	0.006
CP1 vs. CP2	3.79 vs. 4.23	0.6144	0.134	4.576	5	0.006

**Table 5:** Cultural sensitivity parameter.

Cultural sensitivity	Mean score	*β*	SE	*t*	df	*p*
CP1 vs. DL	3.77 vs. 2.07	0.4956	0.150	3.305	5	0.021
CP1 vs. GT	3.77 vs. 1.84	1.0072	0.186	5.402	5	0.003
DL vs. GT	2.07 vs. 1.84	1.5145	0.453	3.345	5	0.02
CP2 vs. DL	4.09 vs. 2.07	0.5306	0.161	3.303	5	0.021
CP2 vs. GT	4.09 vs. 1.84	1.1377	0.117	9.756	5	<0.001
CP1 vs. CP2	3.77 vs. 4.09	0.8830	0.136	6,485	5	0.001

**Table 6:** Persuasiveness parameter.

Persuasiveness	Mean score	*β*	SE	*t*	df	*p*
CP1 vs. DL	4.04 vs. 2.16	0.6703	0.237	2.833	5	0.037
CP1 vs. GT	4.04 vs. 1.89	1.5678	0.273	5.744	5	0.002
DL vs. GT	2.16 vs. 1.89	1.5169	0.421	3.605	5	0.015
CP2 vs. DL	4.39 vs. 2.16	0.6250	0.171	3.650	5	0.015
CP2 vs. GT	4.39 vs. 1.89	1.2966	0.078	16.556	5	<0.001
CP1 vs. CP2	4.04 vs. 4.39	1.1584	0.250	4.641	5	0.006

**Table 7:** Overall performance parameter.

Overall	Mean score	*β*	SE	*t*	df	*p*
CP1 vs. DL	3.87 vs. 2.20	0.7727	0.144	5.353	5	0.003
CP1 vs. GT	3.87 vs. 1.90	0.4667	0.089	5.259	5	0.003
DL vs. GT	2.20 vs. 1.90	0.5667	0.095	5.937	5	0.002
CP2 vs. DL	4.07 vs. 2.20	1.7727	0.198	8.974	5	<0.001
CP2 vs. GT	4.07 vs. 1.90	1.0833	0.100	10.812	5	<0.001
CP1 vs. CP2	3.87 vs. 4.07	0.4416	0.055	8.043	5	<0.001

To assess the consistency and reliability of the seven expert raters’ evaluations, Kendall’s tau correlation matrix was computed (Table 8). The pairwise correlation coefficients range from 0.56 to 0.78, all of which are statistically significant (*p* < 0.05), indicating moderate to strong positive relationships among the raters. Notably, the highest correlation (*τ* = 0.78) is observed between Rater 2 and Rater 5, suggesting a particularly high degree of agreement in their scoring patterns across the four dimensions of fidelity, fluency, cultural sensitivity, and persuasiveness. Even at the lower end of the spectrum (τ ≈ 0.56), the correlations remain sufficiently robust to reflect meaningful concordance among the raters. These findings underscore the presence of a consistent evaluative framework, bolstering confidence in the aggregated scores used to compare translation quality across CP1, CP2, DL, and GT. High inter-rater agreement minimizes the influence of individual bias, thereby enhancing the credibility of the comparative performance metrics. Overall, the moderate to strong correlations affirm that all raters employed comparable evaluative criteria, lending further validity to the subsequent analyses of each system’s translation performance.

**Table 8:** Kendall’s tau correlation matrix.

Rater no.	1	2	3	4	5	6	7
1	1.000	0.560	0.679	0.628	0.699	0.627	0.594
2	0.560	1.000	0.659	0.613	0.597	0.634	0.623
3	0.679	0.659	1.000	0.738	0.699	0.779	0.632
4	0.628	0.613	0.738	1.000	0.594	0.586	0.723
5	0.699	0.597	0.699	0.594	1.000	0.728	0.663
6	0.627	0.634	0.779	0.586	0.728	1.000	0.656
7	0.594	0.623	0.632	0.723	0.663	0.656	1.000

### 4.1 RQ1: performances comparison

**Figure 1:**
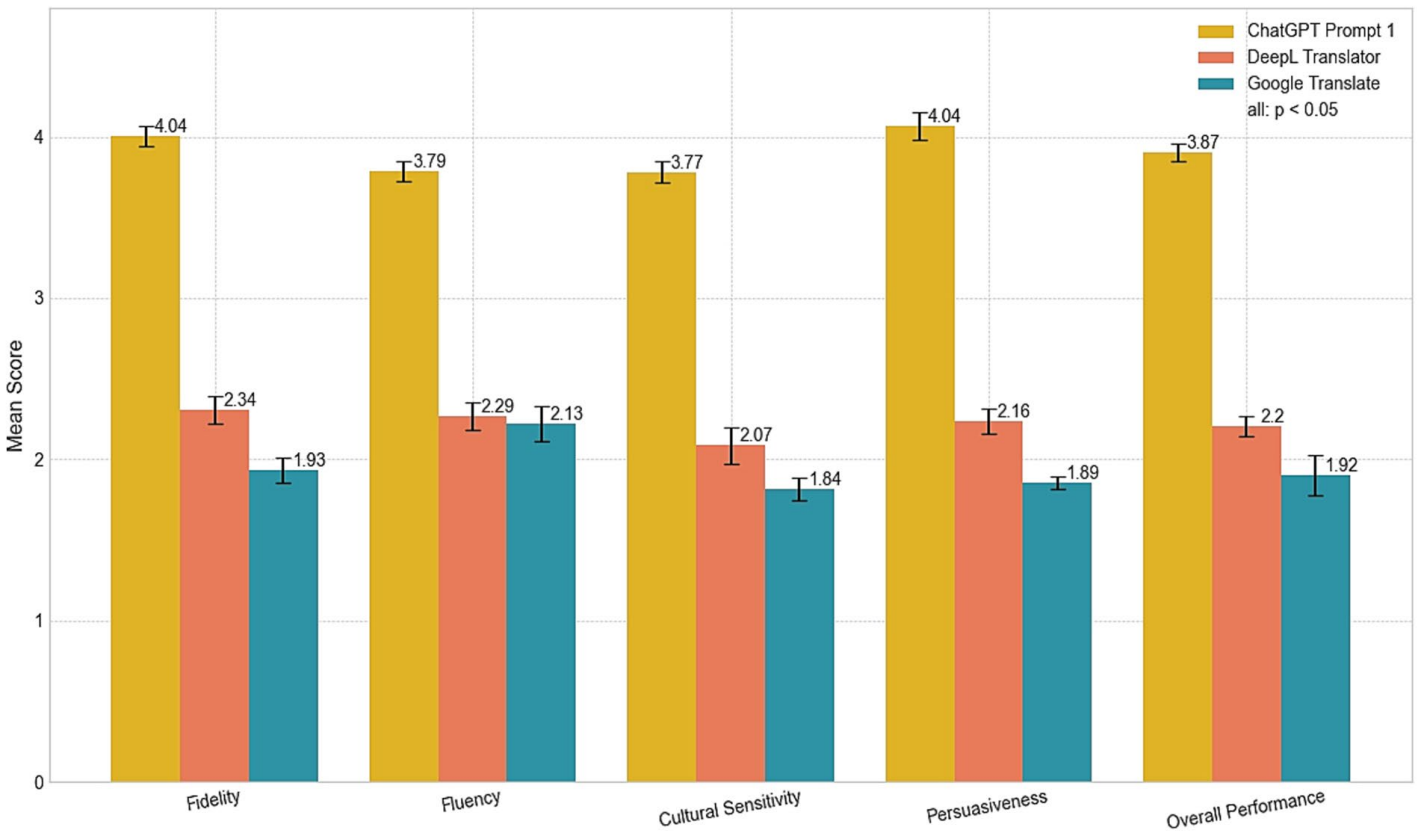
Comparison of translation systems across metrics.

For the first research question, CP2 was excluded from the analysis. As illustrated in Table 2 and Figure 1, the results consistently indicate that CP1 outperformed both DL and GT across all evaluation criteria.

In terms of fidelity, CP1 achieved the highest mean score (M = 4.04, SD = 0.19), indicating a robust capability to preserve the original meaning and intent. In contrast, DL (M = 2.34, SD = 0.26) and GT (M = 1.93, SD = 0.21) exhibited significantly lower fidelity, with issues such as inaccuracies and overly literal translations. For example, the sentence “豫园始建于明嘉靖年间, 最初是四川布政使潘允端的私家园林” (transliteration: yù yuán shǐ jiàn yú míng jiā jìng nián jiān, zuì chū shì sì chuān bù zhèng shǐ pān yǔn duān de sī jiā yuán lín) was translated by CP1 as, “Yu Garden was originally constructed during the Ming Dynasty under the reign of Emperor Jiajing, and it was initially a private garden owned by Pan Yunduan, the Governor of Sichuan.” This version effectively maintains the historical context and formal tone. In comparison, DL’s version—“The Yuyuan Garden was built during the Jiajing period of the Ming Dynasty, and was originally the private garden of Pan Yunduan, the Minister of Sichuan Province”—introduces an error by substituting “Minister” for “Governor,” thereby altering the original meaning. Google Translate’s rendition—“Yu Garden was first built during the Jiajing period of the Ming Dynasty. It was originally the private garden of Pan Yunduan, the governor of Sichuan”—approximates CP1’s output but simplifies certain contextual details and diminishes the formal tone. These differences underscore CP1’s superior contextual understanding and its ability to preserve nuanced meanings relative to its counterparts.

However, despite achieving the highest mean score, CP1 still has room for improvement. Specifically, cultural references such as “明嘉靖年间” (the Jiajing period of the Ming Dynasty) and “布政使” (Bu Zhengshi, a historical governmental title) may require further explanation to enhance comprehension for target foreign audiences and tourists who may not be familiar with these terms. This highlights a fundamental tension in translation: the choice between preserving the original’s terminological authenticity and providing immediate accessibility for the target reader. CP1’s current approach prioritizes a seamless, unannotated reading experience, which, while fluent, risks obscuring the rich historical specificity embedded in the source text. Providing brief annotations or contextual explanations for such cultural references could improve the overall accessibility and informativeness of the translations. For instance, elucidating that “明嘉靖年间” refers to a specific era within the Ming Dynasty and that “布政使” was a high-ranking official responsible for administrative duties in a province, would offer valuable context that preserves the depth and richness of the original text. These findings align with previous studies indicating that traditional machine translation systems often struggle with culturally rich texts (Shao et al., 2018; Huang et al., 2021; Costa-jussà et al., 2023). By contrast, CP’s performance highlights the advancement of neural models in comprehending and preserving nuanced meanings, in line with findings by Vardaro et al. (2019), who reported CP’s ability to generate context-aware translations.

The dimension of fluency further distinguished CP1 from DL and GT, with CP1 receiving a mean fluency score of M = 3.79 (SD = 0.20). By comparison, DL (M = 2.29, SD = 0.23) and GT (M = 2.13, SD = 0.32) exhibited lower fluency, often producing phrasing and sentence structures that felt mechanical or awkward. For instance, in translating the sentence “唐朝人曾说‘蜀中多仙山, 峨眉邈难匹’。不过对于21世纪人, 探访雪山才够范儿” (transliteration: táng cháo rén céng shuō “shǔ zhōng duō xiān shān, é méi miǎo nán pǐ,” bù guò duì yú 21 shì jì rén, tàn fǎng xuě shān cái gòu fàn ér), CP1 rendered it as, “As the Tang Dynasty poets once said, “Sichuan is home to many immortal mountains, but none can rival the distant grandeur of Mount Emei.” However, for the 21st-century traveler, there’s nothing quite like exploring the towering snow-capped peaks of the Greater Snow Mountains.” This translation maintains a natural flow and incorporates stylistic elements appropriate for the target audience, ensuring a smooth and engaging reading experience. In contrast, DL produced a less coherent version: “The Tang Dynasty once said, ‘there are many immortal mountains in Shu, and Emei is hard to match.’ But for the 21st century, visiting snow-capped mountains is the way to go.” While functionally accurate, the phrasing is stilted and lacks the refined style characteristic of persuasive tourism texts. (e.g., translating “才够范儿” merely as “is the way to go”). GT, on the other hand, generated an even less fluent translation: “People in the Tang Dynasty once said, “There are many fairy mountains in Shu, and Emei Mountain is unmatched.” However, for people in the 21st century, visiting snow-capped mountains is the most fashionable.” The phrasing here is mechanical and unnatural, with literal interpretations like “most fashionable” failing to resonate with the intended context of adventurous exploration. Overall, while DL showed modest improvements over GT in avoiding structural rigidity, both were less effective than CP1 in maintaining the refined, persuasive style characteristic of high-quality tourism texts.

While these observations demonstrate CP1’s ability to generate fluent translations, it is crucial to recognize that an independent human translator baseline was not included in this study. The absence of such a benchmark is a significant methodological limitation, preventing a definitive assessment of whether CP1’s fluency meets professional human standards. For example, the classical poetry “蜀中多仙山, 峨眉邈难匹” was translated by CP1 in a manner that maintains a natural flow and incorporates appropriate stylistic elements for the target audience. Nonetheless, this fluency comes at a cost, as the intrinsic beauty and poetic nuance embedded in the original Chinese text were somewhat diminished. While the translation accurately conveys the literal meaning, it fails to capture the lyrical and aesthetic qualities that are characteristic of classical Chinese poetry (Gao R. et al., 2024). This is a classic example of “domestication,” where the foreignness of the source text is smoothed over to conform to the expectations of the target language, potentially sacrificing cultural authenticity for readability. These observations support research by Mohamadi et al. (2023), highlighting how LLMs such as CP leverage broader contextual understanding and advanced neural architectures to produce more fluent and contextually coherent translations. Traditional neural machine translation systems, including DL and GT, often rely on more rigid token-to-token approaches, contributing to awkward phrasing and inconsistent sentence structures. However, even CP1’s superior fluency does not guarantee it will always match human translators in stylistic appropriateness, especially when dealing with highly specialized or poetic text. Without a professional human translation as a reference, we can only conclude that CP1 is more fluent relative to other machines, not that it has achieved a level of fluency that satisfies the nuanced requirements of literary or high-stakes commercial translation. While the system’s broad training corpus allows it to generate coherent sentences, adapting to unique or emerging discourse patterns within specific cultural contexts remains an ongoing challenge. While these observations demonstrate CP’s ability to generate fluent translations, it is important to note that an independent human translator baseline was not included in this study. The absence of such a baseline limits our ability to fully assess whether CP’s fluency and cultural sensitivity meet professional human translator expectations, particularly in handling complex cultural nuances or poetic text. Even though CP’s translations appear “near-human” in terms of fluency, without a professional human translation baseline, we cannot claim that its performance truly matches that of a skilled human translator. This omission is a significant limitation of the current study, and future research should include human translator evaluations to provide a more comprehensive comparison.

Cultural sensitivity scores reflect the ability to adapt idiomatic expressions and culturally embedded meanings. CP1’s mean score of M = 3.77 (SD = 0.18) exceeded those of DL (M = 2.07, SD = 0.31) and GT (M = 1.84, SD = 0.17). Although CP1 demonstrates stronger performance, it remains below the “good” (4.0) and “excellent” (5.0) thresholds, suggesting room for improvement. One illustrative example is the translation of Qingyang Palace [青羊宫被誉为“川西第一道观” (transliteration: qīng yáng gōng bèi yù wéi “chuān xī dì yī dào guàn”)], which CP1 rendered as “Qingyang Palace is renowned as the “First Daoist Temple in Western Sichuan” and is the oldest and largest Daoist temple still standing in Chengdu.” This translation effectively maintains the formal tone and cultural significance. In contrast, DL produced a more literal version: “Qingyang Palace is known as “the first Taoist temple in western Sichuan” and is the oldest and largest Taoist temple in Chengdu.” While functional, the phrase “the first Taoist temple in western Sichuan” lacks the refined adaptation required to resonate with the cultural and historical significance of the original. Google Translate’s version, “Qingyang Palace is known as the ‘No. 1 Taoist Temple in Western Sichuan,’” simplifies the expression further and introduces colloquial phrasing like “No. 1,” which detracts from the cultural sophistication expected in tourism texts.

Furthermore, CP’s translations of culturally rich tourism content, such as descriptions of local festivals, cuisines, and traditional ceremonies, occasionally oversimplify culturally specific details or overly emphasize visually appealing elements at the expense of historical and symbolic depth. For instance, while CP translated “青羊肆” as “the Qingyang market of Chengdu,” it did not adequately communicate the cultural and historical significance of the location as a symbolic link to Laozi’s journey. Furthermore, CP1’s cultural sensitivity score remains below the “good” threshold, indicating that the system requires improvements in conveying nuanced cultural connotations—including local customs, symbolic imagery, and socio-historical references—which are essential for engaging an international audience in tourism translation. These findings are consistent with earlier critiques, such as those by Nazir and Wang (2023), which highlighted the challenges machine translation systems face when dealing with intricate layers of cultural meaning. The relatively low variability in CP1’s scores (SD = 0.18) suggests that its performance, while consistently better than DL and GT, lacks the flexibility and depth needed to address the most culturally nuanced content. However, to improve cultural sensitivity, more concrete strategies can be employed. For instance, CP could be fine-tuned on annotated corpora of classical Chinese tourism writing to better capture the nuances and traditional cultural references often embedded in such texts. Additionally, translations could include brief explanatory parentheticals to offer deeper context and historical insight for international readers (Li et al., 2024). This would allow for a richer understanding of the cultural and historical layers of the original text. Furthermore, integrating external cultural knowledge bases (e.g., Wikidata) at inference time could help provide real-time access to detailed cultural information, enhancing ChatGPT’s ability to incorporate relevant cultural context and avoid oversimplifications or inaccuracies. These strategies, including fine-tuning on specialized corpora, adding explanatory context, and integrating external knowledge, provide a clear pathway toward improving the system’s cultural adaptability.

The dimension of persuasiveness emerged as a key differentiator. CP1 attained a strong persuasiveness score (M = 4.04, SD = 0.26), surpassing DL (M = 2.16, SD = 0.28) and GT (M = 1.89, SD = 0.16). This strength reflects CP1’s ability to preserve the emotional and promotional tone critical in tourism materials. For example, in translating the description of Chongqing’s karst landscapes [“当人们流连于千姿百态的峰丛, 石林和溶洞等喀斯特风景时, 在重庆的地底却深藏着世界级别的喀斯特地貌群, 它们在近年才被中外探险家揭开神秘面纱” (transliteration: “dāng rén men liú lián yú qiān zī bǎi tài de fēng cóng, shí lín hé róng dòng děng kā sī tè fēng jǐng shí, zài chóng qìng de dì dǐ què shēn cáng zhe shì jiè jí bié de kā sī tè dì mào qún, tā men zài jìn nián cái bèi zhōng wài tàn xiǎn jiā jiē kāi shén mì miàn shā”)], CP1 rendered it as, “While people marvel at the diverse karst landscapes of peaks, stone forests, and limestone caves, beneath the city of Chongqing lies a world-class karst geological wonder that has only recently been uncovered by explorers from both China and abroad.” This translation not only conveys the grandeur of the landscape but also employs evocative language to engage readers and evoke a sense of adventure. Moreover, it balances descriptive detail with a promotional call to action, a hallmark of effective tourism translation.

DL rendered the passage as, “While people linger over the karst landscapes of peaks, rock forests, and caves, there are world-class karst formations hidden deep beneath the ground in Chongqing, which have only been unveiled by Chinese and foreign explorers in recent years.” Although this version is accurate, it lacks the promotional rhetoric necessary to engage readers. The expression “linger over the karst landscapes” is functional but does not evoke the same emotional appeal as CP1’s “marvel at the diverse karst landscapes.” Moreover, literal phrases such as “formations hidden deep beneath the ground” fail to capture the excitement and mystique of the original text. Overall, DL’s focus on literal accuracy over stylistic enhancement contributes to its lower persuasiveness score and reflects a limited adaptation to the marketing tone typical of tourism texts.

GT’s output is notably less engaging. For instance, it’s rendering—“While people are lingering in the karst landscapes of various shapes and sizes, such as peaks, stone forests and caves, there are world-class karst landforms hidden deep underground in Chongqing, which have only been unveiled by Chinese and foreign explorers in recent years”—lacks the dynamism required for effective tourism marketing. The phrase “lingering in the karst landscapes of various shapes and sizes” appears mechanical and uninspired, while “hidden deep underground in Chongqing” is direct but fails to evoke the curiosity and emotional resonance necessary to attract international tourists. Although GT accurately conveys technical information, its limited stylistic sophistication and cultural adaptation diminish its promotional impact. These shortcomings indicate that systems like GT require more advanced contextual and rhetorical modeling to address the nuanced demands of tourism texts. Conversely, while CP1 successfully injects wonder and engagement, it carries a risk of over-embellishment or omitting critical information vital for informed tourist decision-making. Overall, CP1 outperforms both DL and GT regarding fidelity, fluency, cultural sensitivity, and persuasiveness. This finding concurs with broader research suggesting that large language models, supported by extensive training data and advanced neural architectures, yield more contextually sensitive and rhetorically effective translations for complex genres such as tourism.

### 4.2 RQ2: impact of tailored prompts

**Figure 2:**
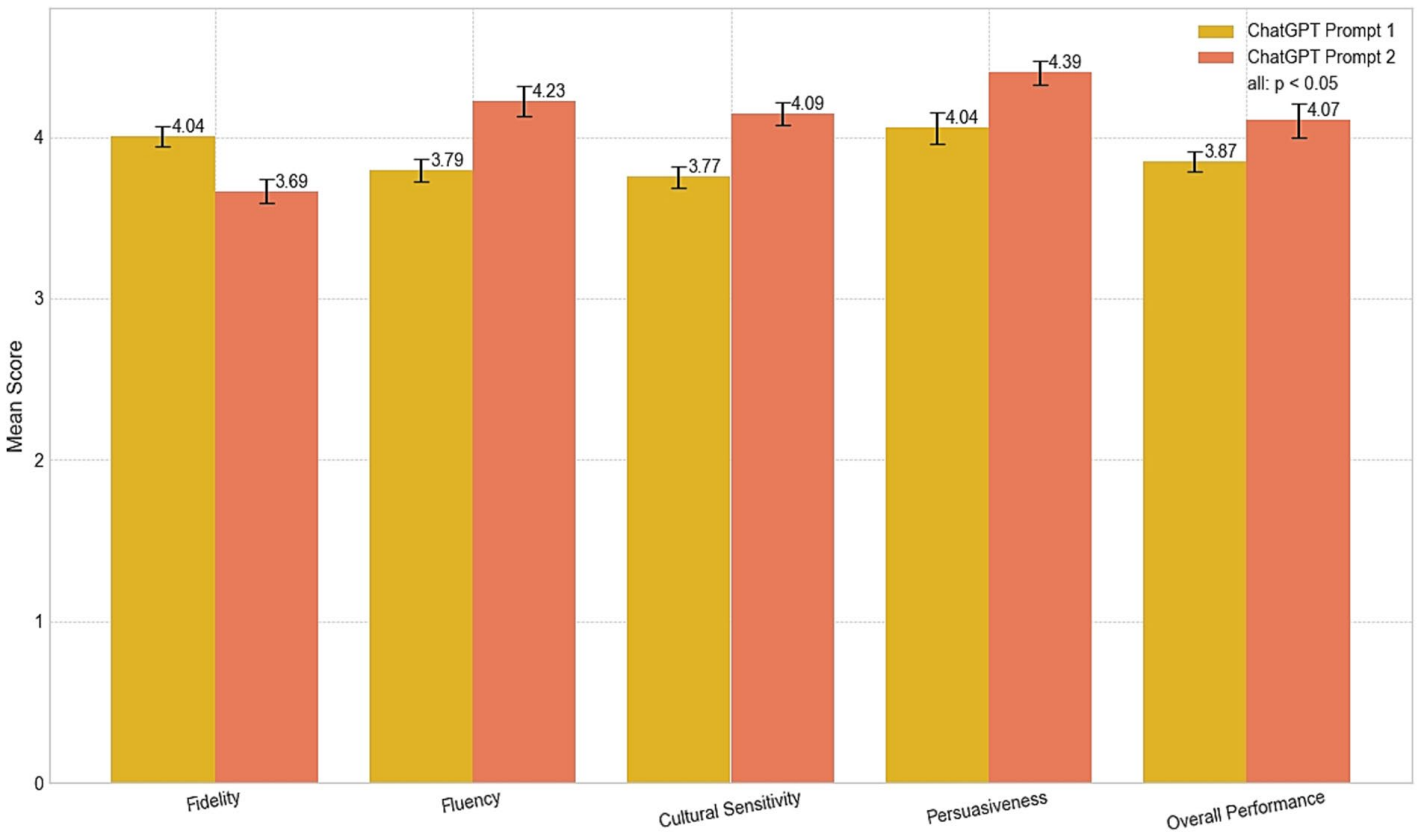
Performance comparison of ChatGPT prompts.

The comparison (see Table 2 and Figure 2) between CP1 and CP2 demonstrated the significant impact of tailored instructions in enabling the model to address the nuanced demands of tourism-related texts. CP2, which utilized tailored instructions to interpret the source text and emphasize culturally sensitive, persuasive language, demonstrated marked improvements over CP1. Specifically, while CP1 achieved strong baseline scores in fidelity (M = 4.04, SD = 0.19), fluency (M = 3.79, SD = 0.20), cultural sensitivity (M = 3.77, SD = 0.18), persuasiveness (M = 4.04, SD = 0.26), and overall performance (M = 3.87, SD = 0.15), CP2 outperformed it in fluency (M = 4.23, SD = 0.29), cultural sensitivity (M = 4.09, SD = 0.20), persuasiveness (M = 4.39, SD = 0.20), and overall performance (M = 4.07, SD = 0.32). These findings underscore the significance of prompt engineering in enhancing translation outputs, producing texts that are more grammatically coherent, contextually nuanced, and culturally resonant qualities that are essential for effective communication in the tourism sector.

**Figure 3:**
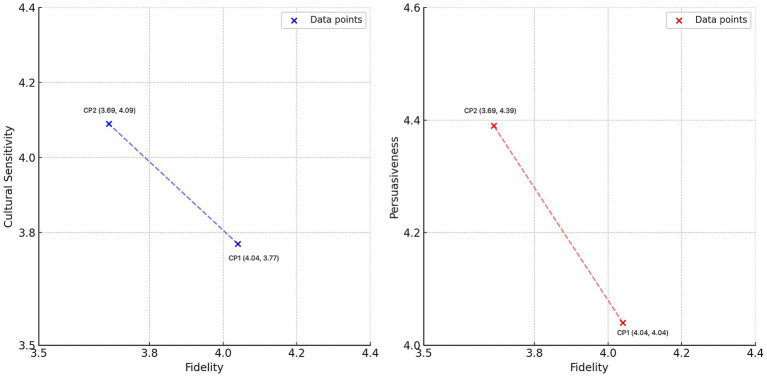
Fidelity vs. cultural sensitivity and fidelity vs. persuasiveness.

However, the advancement of stylistic and rhetorical enhancements appear to compromise fidelity—the degree to which a translation preserves the original meaning. In our study, fidelity in CP2 (M = 3.69, SD = 0.22) was slightly lower than in CP1 (M = 4.04, SD = 0.19). This apparent compromise invites a critical re-examination of the concept of fidelity itself. While traditionally defined by literal, semantic accuracy, fidelity in a persuasive context might be more meaningfully measured by its alignment with the source text’s pragmatic intent or rhetorical effect. This tension is vividly illustrated by a translation example concerning Du Fu’s Thatched Cottage [让草堂从此走入中国文学史 (transliteration: ràng cǎo táng cóng cǐ zǒu rù zhōng guó wén xué shǐ)]. CP1 rendered the phrase as “making the cottage an important landmark in Chinese literary history,” whereas Prompt 2 rephrased it as “immortalizing the site in Chinese literary history.” However, framing this as a simple trade-off between accuracy and creativity may be reductive. The term “immortalizing,” while not a literal translation, arguably achieves a higher form of fidelity to the source text’s cultural significance and its intended emotional resonance. The interpretive liberty taken by CP2 could thus be seen not as a failure of fidelity, but as a successful transfer of rhetorical function, aligning with the findings of Gao Y. et al. (2024) on the challenges of balancing these elements. As further illustrated in Figure 3, our analysis reveals a significant inverse relationship between literal fidelity and the communicative metrics of cultural sensitivity and persuasiveness. A comparison between the outputs of two prompts, CP1 and CP2, demonstrates a clear inverse relationship. Specifically, while CP1 achieved a higher fidelity score (M = 4.04), it registered lower scores in cultural sensitivity (M = 3.77) and persuasiveness (M = 4.04). Conversely, CP2 yielded superior performance in cultural sensitivity (M = 4.09) and persuasiveness (M = 4.39), but this came at the expense of reduced fidelity (M = 3.69). This dialectic suggests that for certain communicative goals, a decrease in one form of fidelity (literal) is a necessary precondition for an increase in another (pragmatic and cultural). For instance, whereas CP1 rendered the gardens surrounding Du Fu’s Thatched Cottage as “a peaceful retreat,” CP2 transformed it into “a perfect escape into natural beauty and cultural reverence.” This example underscores the transformative potential of prompt engineering, yet it also exposes the subjective nature of these enhancements; what one reader perceives as “resonant,” another might view as “embellished” or an “interpretative overreach.”

Ultimately, these findings shift the discourse from a simple question of “which prompt is better?” to a more nuanced inquiry: “What theory of translation is being embedded in a given prompt, and how does it align with the text’s specific communicative goal (skopos)?” The observed reduction in literal fidelity is not merely a technical limitation but a reflection of a deliberate, albeit automated, choice to prioritize communicative effect. This necessitates a more sophisticated approach to prompt design, moving beyond static instructions toward dynamic strategies that might, for instance, specify which parts of a text demand strict fidelity and which permit creative adaptation. The challenge for future research and practice is therefore not simply to balance these competing demands, but to cultivate a critical awareness—both in human translators and potentially in the models themselves—of when and why to privilege one translational priority over another. This requires careful calibration to avoid excessive interpretive liberties while still harnessing the model’s capacity to create truly engaging and culturally resonant tourism texts.

### 4.3 RQ3: advantages and limitations of ChatGPT

CP demonstrates notable strengths and limitations in the field of tourism texts translations. When compared to traditional systems like GT and DL, CP produces natural, engaging, and contextually appropriate translations. It consistently generates fluent and grammatically coherent output that often mirrors human-quality work, yielding superior fluency scores. As Aycock and Bawden (2024) note, advanced neural language models excel in context awareness and grammatical accuracy, a finding that aligns with CP’s performance in this study. Its ability to produce polished, reader-friendly texts is particularly beneficial in tourism translation, where natural language enhances readability and audience engagement. In contrast, GT often struggles with coherence in complex sentences, and DL occasionally produces awkward phrasing.

CP exhibits a notable capacity for cultural sensitivity in tourism translations by rendering culturally significant expressions into idiomatic, audience-friendly equivalents. However, this practice is not without its own set of ethical and hermeneutic challenges. This approach, while seemingly beneficial for audience engagement, contrasts with traditional systems that tend to translate cultural references literally (AlAfnan, 2024). Nevertheless, ChatGPT sometimes compromises the original meaning of culturally specific elements to achieve a more rhetorically compelling output, underscoring the need for human oversight to maintain fidelity. This raises a critical question: is this an act of genuine cultural brokerage, or a form of semantic gentrification where nuanced cultural elements are flattened for easier consumption by the target audience? Similar challenges in balancing cultural resonance and fidelity have been noted in prior studies (Zaid and Bennoudi, 2023; Yao, 2024). In terms of persuasiveness, CP’s ability to produce engaging and emotionally resonant translations enhances the marketing appeal of tourism texts. This finding corroborates earlier research highlighting the potential of large language models to mimic human creativity in their outputs (Organisciak et al., 2023; Franceschelli and Musolesi, 2024).

However, CP occasionally prioritizes rhetorical effectiveness over fidelity, at times resulting in fabricated or overly interpretive content. This phenomenon, often termed “hallucination,” is more than a mere technical glitch; it points to a fundamental limitation in the model’s epistemological reliability, as it is optimized for probabilistic coherence rather than factual verification. This trade-off reveals a broader limitation: preserving the original meaning and intent of the source text. Despite its strengths in fluency and cultural sensitivity, CP’s average fidelity score fell between “moderate” and “good,” indicating persistent difficulties in preserving the original meaning and intent of the source text. This aligns with observations by Williamson and Prybutok (2024), who discuss “hallucination” in AI-generated outputs, where the model introduces inferred or fabricated elements that compromise accuracy. In the previously mentioned example, the translation of “Yu Garden” highlighted this issue. The source text described its construction and its turbulent history. While CP1 adhered more closely to the original description, stating that “the garden underwent several large-scale restorations, gradually restoring its former splendor,” CP2 introduced a fabricated element by elaborating, “Today, Yuyuan Garden stands as a stunning example of classical Chinese garden design, offering visitors a glimpse into Shanghai’s rich history and the resilience of its cultural heritage. Whether you are drawn to its peaceful ambiance or its fascinating past, a visit to Yuyuan Garden is an unforgettable journey through time.” While such embellishments enhance rhetorical appeal, they distort the original content—a problem less prevalent in systems like DL that prioritize factual precision over stylistic enhancement, highlighting a fundamental trade-off between engaging rhetoric and verifiable truth.

CP’s performance improvements through tailored prompt engineering—exemplified by Prompt 2—come with inherent limitations. Although refined prompts enhance fluency, cultural sensitivity, and persuasiveness, they also introduce variability tied to prompt quality. As noted by Henrickson and Meroño-Peñuela (2023), optimal performance in advanced models depends on precise instructions; without such carefully designed prompts, CP reverts to near-baseline output, which, despite outperforming traditional systems, does not fully utilize its potential. This dependence on specialized expertise limits accessibility for those without adequate resources for effective prompt development. This introduces a potential new form of digital divide within the translation field, where practitioners with advanced prompt engineering skills can elicit superior results, while others may be left with inconsistent or suboptimal outputs, thereby creating a new hierarchy of technological competence.

The obtained data underscores CP’s relative strengths across key dimensions of tourism translation. CP2 achieved an overall performance score of 4.07 compared to CP1’s 3.87, reflecting improvements in fluency, cultural sensitivity, and persuasiveness. However, the fidelity scores for CP2 (3.69) highlight ongoing challenges in preserving the original meaning of source texts. These findings affirm the importance of human oversight in addressing fidelity issues, particularly in high-stakes or culturally sensitive contexts. While this symbiotic relationship is often framed optimistically, it warrants a more critical examination. While CP’s outputs are suitable for drafting compelling tourism content, the human translator’s role remains indispensable for bridging identified gaps in CP’s outputs. This collaborative model positions CP as a valuable tool for producing initial drafts, freeing human translators to focus on tasks requiring higher-level expertise. However, an alternative perspective suggests this could lead to the deskilling of the translation profession, relegating human experts to the role of post-editors and potentially devaluing the nuanced cognitive processes that define high-quality translation. The long-term sustainability of this human-AI partnership, and its impact on the professional identity and economic viability of translators, thus remains an open and crucial question for future research.

## 5 Discussion

The findings indicate that CP consistently outperformed GT and DL in fidelity, fluency, cultural sensitivity, and persuasiveness, delivering outputs that were more engaging, rhetorically effective, and culturally attuned. Although tailored prompts enhanced fluency, cultural adaptation, and persuasive impact by reinterpreting idiomatic expressions and cultural references, they occasionally introduced interpretative elements that deviated from the original meaning, highlighting a tension between textual accuracy and cultural resonance.

Traditional systems, while generally producing functional translations, struggled with the stylistic and cultural nuances characteristic of tourism discourse. GT typically rendered overly literal outputs lacking both fluency and cultural adaptation, whereas DL, despite moderate gains in fluency, was limited in conveying rhetorical and promotional elements. Conversely, CP’s advanced language modeling achieved translations more closely aligned with communicative and promotional objectives, though its performance remained contingent on prompt engineering and sometimes resulted in fabricated content to enhance rhetorical effect. It is important to temper the generalization of these findings to other language pairs. The unique aspects of Chinese—such as its logographic script, syntactic differences, and cultural references—may not necessarily translate to other language combinations. While the study highlights the promising results of LLM-based translation systems in tourism contexts, it cannot claim that the same outcomes will apply universally across different languages or domains. To validate the consistency of these findings, future research should explore translation performance in different language pairs, such as Spanish-to-English tourism, Arabic-to-English, or French-to-English, to test whether similar trends hold in other linguistic and cultural settings.

Expanding upon these findings, the real-world implications of LLM translation systems for various stakeholders should be considered. For translation teams, the use of LLMs like CP could be integrated into workflows as a tool for generating initial drafts, allowing human translators to focus on high-value revisions that require deeper cultural insights or subject-specific expertise. This approach would harness the speed and scalability of AI while ensuring the accuracy and nuance necessary for professional translations. For tourism marketers, LLMs can be leveraged to create multilingual websites quickly and cost-effectively. However, to avoid cultural missteps, it would be essential to establish guardrails that flag potentially problematic cultural references, idiomatic expressions, or inaccuracies, ensuring that the final content resonates with diverse international audiences. For technology developers, there is an opportunity to design domain-adaptive translation interfaces that automatically flag potential fidelity issues. Such systems could enhance the user experience by highlighting instances where translations may have deviated from the original meaning, thus enabling human editors to make timely adjustments. These innovations would support the deployment of AI-driven translation tools in real-world applications while addressing the specific needs of tourism translation. By addressing these practical considerations, we can better understand how AI translation tools like CP can complement and enhance human workflows in real-world translation environments, particularly within the tourism industry.

## 6 Conclusion

This This study conducted a systematic, multi-dimensional evaluation of ChatGPT (CP) in comparison to Google Translate (GT) and DeepL (DL) in the domain of Chinese-to-English tourism translation. The results demonstrate CP’s notable strengths in generating fluent, persuasive, and culturally aware translations, particularly when guided by task-specific prompts. These findings suggest that ChatGPT’s ability to adapt through prompt engineering holds significant potential beyond tourism translation, with applications in content localization, creative writing, and cross-cultural communication. Importantly, this study illuminates the nuanced trade-offs between fidelity on one hand, and cultural sensitivity and persuasiveness on the other. While tailored prompts can enhance fluency and rhetorical effectiveness, they may also introduce interpretive liberties that deviate from the original meaning. This fundamental tension underscores a critical takeaway: the integration of AI tools like ChatGPT into professional workflows should complement, rather than replace, human expertise. This is especially true in culturally sensitive or high-stakes contexts where misinterpretation could carry serious consequences. While our study highlights the value of prompt design, it also reveals that even sophisticated prompting cannot fully replicate human judgment. Moreover, as with other large language models (LLMs), ChatGPT remains inherently prone to hallucinations (Hamid, 2024), where fabricated content can be introduced, undermining both fidelity and cultural authenticity. This necessitates vigilant human oversight and critical post-editing to bridge the gap between AI-generated drafts and publishable, trustworthy translations.

Methodologically, this research underscores the value of combining human expert evaluation with prompt-based task control. The integration of metrics for cultural sensitivity and persuasiveness provides a more comprehensive framework for future MT assessments beyond traditional adequacy and fluency scales. However, this study has several limitations. First, its exclusive focus on ChatGPT omits comparison with other powerful LLMs, such as DeepSeek-R1, which may offer alternative trade-offs in performance and transparency (Jin et al., 2025). Second, its scope is restricted to Chinese-to-English translation, which limits the generalizability of the findings to other language pairs, particularly those involving low-resource or typologically diverse languages. Finally, the broader ethical implications of using AI-generated translations in professional and culturally nuanced domains remain unaddressed. Future research should therefore expand these comparative evaluations across multiple LLMs and assess performance in a wider range of multilingual contexts. Further investigation into the effects of domain-specific fine-tuning versus prompt design on translation accuracy and ethical integrity is also warranted. Crucially, empirical comparisons between LLMs and professional human translators remain essential for understanding the evolving role of AI in translation practice. As LLM technology continues to advance, a responsible and effective integration into professional workflows will demand a sophisticated understanding of both its powerful affordances and its inherent limitations.

## Data Availability

The original contributions presented in the study are included in the article/Supplementary material, further inquiries can be directed to the corresponding author.
